# Hepatitis B, Hepatitis C and Human Immunodeficiency Virus Seropositivity Among Children in Kabul, Afghanistan: A Cross-Sectional Study

**DOI:** 10.5812/hepatmon.16154

**Published:** 2014-03-08

**Authors:** Ilhan Asya Tanju, Fatma Levent, Rabia Gonul Sezer, Ferhat Cekmez

**Affiliations:** 1 Department of Pediatrics, Gulhane Military Medical Academy, GATA Haydarpasa Training Hospital, Istanbul, Turkey; 2 Department of Pediatrics, Texas Tech Health Sciences Center, Lubbock, USA; 3 Department of Pediatrics, Zeynep Kamil Maternity and Children’s Diseases Training and Research Hospital, Istanbul, Turkey; 4 Department of Pediatrics, Gulhane Military Medical Academy, School of Medicine, Etlik, Turkey

**Keywords:** *Hepatitis B Virus*, *Hepatovirus*, *HIV*, *Pediatrics*

## Abstract

**Background:**

Hepatitis B virus (HBV), hepatitis C Virus (HCV), and human immunodeficiency virus (HIV) infections are significant causes of morbidity and mortality all over the world, especially in underdeveloped countries like Afghanistan. Limited data are available concerning the seroprevalence of HBV, HCV and HIV in the pediatric age group in Afghanistan .

**Objectives:**

The aim of the study was to assess HBV, HCV and HIV serology among children at an outpatient clinic in Kabul.

**Patients and Methods:**

A total number of 330 children were included to the study from outpatient clinics of Ataturk Kabul ISAF Role II Military Hospital from May to November 2012. Hepatitis B surface antigen (HBsAg), hepatitis C antibody (anti-HCV), and human immunodeficiency virus antibody (anti-HIV) were measured.

**Results:**

The mean age of children was 6.5 ± 4.2 years. The frequency of positive results for HBsAg, anti-HBs and anti-HCV in all age groups were 12 (3.6%), 47 (14.2%) and 2 (0.6%), respectively. Anti-HIV was not detected in any of the children's serum samples. The frequency of positive results for HBsAg was significantly higher in children older than six years than in other age groups.

**Conclusions:**

Vaccination program including HBV has begun during the last five years in Afghanistan. The continuation of the vaccination program is of great importance. Vaccination program and implementation steps should be revised and the deficiencies, if any, should be overcome without delay.

## 1. Background

Hepatitis B Virus (HBV), hepatitis C Virus (HCV), and human immunodeficiency virus (HIV) infections are significant causes of morbidity and mortality all over the world (1, 2). These infections are even more important health problems in underdeveloped countries, especially Afghanistan, which is the second least developed country based on the human development index of 177 countries (3, 4). HBV is endemic in South East Asia, including Afghanistan (5). Afghanistan would be at a significant risk with the increasing seropositivity of these viruses. There is limited epidemiological data about infectious diseases in Afghanistan and the most of the available data are from specialized groups such as intravenous drug users, sex workers, and intrapartum women (6-11). There is limited data concerning the seroprevalence of HBV, HCV and HIV in the pediatrics age group in Afghanistan. The studies investigating the pediatrics are the data of Afghan immigrant children living in the neighboring countries. The status of ongoing war for more than 20 years, inadequate health and social structure, low educational level, and socio-political uncertainty are the limitations to epidemiological data collection and analysis in Afghanistan (12).

The aim of this study was determination of the seropositivity of Hepatitis B surface antigen (HBsAg), hepatitis B surface antibody (anti-HBs), hepatitis C antibody (anti-HCV), and human immunodeficiency virus antibody (anti-HIV) in pediatrics evaluated at outpatient clinics of a military hospital in Kabul province of Afghanistan. In addition, after a five-year period of HBV vaccination, we assessed the differences in seropositivity of HBsAg and anti-HBs in children between one to six years of age and those older than six years.

## 2. Objectives

The main idea was to assess HBV, HCV, and HIV seropositivity among children at an outpatient clinic in Kabul.

## 3. Patients and Methods

This outpatient-based cross-sectional study included 330 children from the clinics of Ataturk Kabul ISAF Role II Military Hospital from May through November 2012. The hospital is in Kabul, on the road to Pakistan, within Dogan ISAF Military Camp, Uthil district. It is a general health care center and a military government hospital with 22 beds. The hospital serves a population mostly from Kabul and the suburbs. The residential area around the military camp and the military hospital has a lower socioeconomic and educational level than the Kabul city center. Ethnic origin of people settling around the military camp is mostly consisted of Pashtuns. Patients from the outpatient clinics were included in the study, and the inclusion criteria were defined as follows: patients of either gender aged 1 to 16. Patients with a history of operation, blood transfusion, jaundice, or hepatitis were not included in the study. All eligible patients were examined and blood samples were obtained at the time of evaluation. HBsAg, anti-HBs, anti-HCV and anti-HIV tests were performed. Hepatitis B core antibody (anti-Hbc) was not evaluated since its proper kit was not available. The serum samples were analyzed within two hours in the laboratory of Ataturk Kabul ISAF Role II Military Hospital. The samples were studied with fast cassette kits (Laboquick kits, codes: LBAC.01, LBAB.01, LBHC.01, HIV1/2, manufacturer: Koroglu Medical Devices, Izmir, Turkey). Samples were examined according to the manufacturer’s instructions. The patients were informed about the results providing their parents the results of the tests. Three doses of HBV vaccination was offered to all HBsAg and anti-HBs negative patients and to the families of HBsAg positive patients. Informed parental consent was obtained from all participants, and the study was approved by the local ethics committee (Islamıc Republic of Afghanistan Ministry of Public Health Instıtutional Review Board 5.5.2012 No: 22531). Considering that nearly two million children were living in Kabul, the sample size of 400 was calculated with 95% confidence interval and 2% standard deviation from the population with this formula:

n = Nt^2^pq/d^2 ^(N-1) + t^2^pq (n = sample size, N = population, t^2 ^= statistical parameter, d^2^ = 2% standard deviation from the population, P = 0.05 incidence of the illness, Q = (t-p) = 0.95). Statistical analysis was performed using the statistical package for social sciences (SPSS version 13.0; SPSS Inc., Chicago, IL, USA). The χ^2^ test was used to evaluate the association between seropositivity rates and the age groups. A P value of < 0.05 was considered statistically significant.

## 4. Results

Finally, 350 children, 157 (44.8%) girls and 193 (55.2%) boys, were enrolled in this study. The mean age of participants was 6.5 ± 4.2 years. Twenty participant were excluded: 9 (% 2.5) due to operation, 6 (% 1.7) previous blood transfusion, and 5 (% 1.4) hematological and oncological diseases. There were 161 (48.8%) children between one to six years of age and 169 (51.2%) children between six and 15 years of age. The HBsAg, anti-HBs, and anti-HCV seropositive results were seen in 12 (3.6%), 47 (14.2%), and 2 (0.6%) children in all age groups, respectively. Anti-HIV positive result was not detected in any of the children (Table 1). 

**Table 1.** The Seropositivity of HBsAg, Anti-HBs, Anti-HCV, and Anti-HIV Tests and the Comparison of Seropositive Results Based on the Children' Age Groups (n = 330) ^a^, ^b^

	HBsAg Positivity	Anti-HBs Positivity	Anti-HCV Positivity	Anti-HIV Positivity
**Participants’ age**	3.6 (12)	14.2 (47)	0.6 (2)	0
1- < 6	0.6 (1)	19.3 (31)	0	0
6-15	6.5 (11)	9.5 (16)	1.2 (2)	0
**P value**	0.004	0.011	> 0.05	NA

^a^ Abbreviation: NA, not applicable.

^b^ Data are presented in No. (%).

HBsAg positivity was significantly higher in children older than six years (P = 0.004). HBsAg positivity was seen in one out of 161 (0.6%) children between one to six years of age, whereas it was 11 out of 169 (6.5%) children between six to 15 years of age. Anti-HBs positive results were significantly lower in the children between six to 15 years of age in contrast to the younger children (P = 0.011). Anti-HBs positive results were present in 31 (19.3%) children in the age group one to six and 16 (9.5%) children in the age group six to 15 years. There was no significant difference between the age groups with regard to anti-HCV positive results; two positive patients were in the age group six to 15 years. The HBsAg positivity was significantly higher in girls than in boys of six to 15 years of age group (P = 0.016).

## 5. Discussion

Hepatitis B virus, HCV and HIV continue to be significant causes of morbidity and mortality all over the world and are endemic in South East Asia including Afghanistan (1). The limited epidemiological data on these diseases have been obtained either from specific groups or from Afghan immigrants living in neighboring countries such as Iran and Pakistan. Vaccination program including HBV has begun within the last five years in Afghanistan. According to WHO the rate of vaccination with three doses of HBV vaccine in children younger than one-year was 85% in 2008 and 83% in 2009 (1). Despite the socioeconomic condition of Afghanistan, transition to vaccination program for an endemic disease and the vaccination rates seem promising. The results of our study reflected the effects of the vaccination program. In this study, HBsAg seropositive results were 0.6% for the children between one and six years of age, whereas the rate was 6.5% in children between six to 15 years of age. Similarly, anti-HBs seropositive results were significantly higher in children between one and six years of age (19.3%) than in children between six to 15 years of age (9.5%). This significant difference between children between one and six years of age and children older than six years might be a result of the vaccination program. There was not any information available about the vaccination schedule of our patients because of the low literacy rate of the parents. Even if the vaccination was done, no document related to the name or dose of the vaccines was available.

The prevalence of HBsAg in limited and specific groups of Afghans has been reported between 1.23% and 8.3% (6-11). In studies from Pakistan, the prevalence of hepatitis B infections is between 1.7% and 5.5% in the children (13). There is no data available concerning the prevalence of HIV or HCV in Pakistan or Afghanistan. Studies on the prevalence of HBsAg in Afghanistan did not reflect the entire population as they included specific populations such as sex workers, intrapartum women, and intravenous drug users, with an incidence rate between 1.5% and 6.5% (7-9, 14). High values are reported especially from the Afghan refugee camps in neighboring countries. The prevalence rate of HBsAg in Afghan immigrants in Balochistan of Pakistan was 8.3% (10). The same study reported the incidence of HBsAg between the ages of three to 23 years as 5.6% out of 301 patients (10). Since 2001, three million immigrants returned back to Afghanistan, mainly to Kabul. Since more people are planning to return, this might be considered as a significant risk for the country. The Central Blood Bank in Kabul reported the HBsAg frequency in donors as 3.9% from March to December 2006 (6). In our study, the frequency of HBsAg was 3.6% between the ages of one to-15 years. The only study including the same age group was from Balochistan camp by Quddus et al. with a prevalence rate of 5.6% (10). Our lower prevalence might be related to the age group, higher rate of horizontal transmission in the camp area, and fully vaccination with three doses of HBV vaccine in only 1% of children in the area. The studies among special populations with higher risk factors had, as predicted, higher rate of seropositive results. Todd et al. (7) reported HBsAg prevalence 1.53% in 4452 intrapartum women and they related this low rate to the higher incidence of HBsAg carriage in men and to sexual transmission. The present study results were similar to the results of Central Blood Bank of Kabul. The increase in drug users, unhealthy injection practices, the return of immigrants back to Afghanistan, and the ongoing war over a decade are the major concerns about the increase in the HBsAg prevalence. The studies on Afghan population reported anti-HCV prevalence rate between 0.3% and 36.4% (6,8,9). The rate is high (36%) especially in intravenous drug users (9). The increase in the use of intravenous drugs in Afghanistan is a matter of concern. Many studies reported anti-HCV prevalence as follows: 1.9% by Central Blood Bank of Kabul, 0.3% in obstetric population by Todd et al. report, and 1.92% in sex workers (6, 8, 14, 15). In this study, only 2 (0.6%) children with anti-HCV positive results, were found, in the age group of six to 15 years. Fathers of both children had also anti-HCV positive results. Risk factors for transmission of HCV in childhood are limited and risk of transmission may increase with age. Studies concerning HIV seroprevalence were also similar with an incidence of 0-3%; however, data pertaining to the children population were not clear (6-9, 15). The incidence increases to 3%, especially in intravenous drug users. Similar to HCV, the increase use of intravenous drugs correlates with the higher seropositive results. No anti-HIV positive results were detected in the present study. In our study which can be explained by the small sample size and maybe less intravenous drug users in comparison to the neighboring countries due to lower socioeconomic conditions. One limitation to our study was that it might not reflect the prevalence in all regions of Afghanistan and the sample size was small; however, this was the preliminary study from Afghanistan. As a result, HBV, HCV and HIV are endemic in Kabul province in adults but in children there is not enough data; hence, epidemiological studies with larger populations from different areas of Afghanistan, which might reflect the true status of country regarding these infections prevalence, seem essential.
